# Supplementary material for: Single-cell analysis of Schistosoma mansoni identifies a conserved genetic program controlling germline stem cell fate
Source: Nat Commun. 2021 Jan 20;12:485. doi: 10.1038/s41467-020-20794-w (PMC7817839; doi:10.1038/s41467-020-20794-w)
Supplement: Supplementary file 1 — Supplementary Information [file 41467_2020_20794_MOESM1_ESM.pdf]

Supplementary Information

Single-cell analysis of *Schistosoma mansoni* identifies a conserved genetic program controlling germline stem cell fate

Pengyang Li[1], Dania Nanes Sarfati[2,#], Yuan Xue[1,#], Xi Yu[1], Alexander J. Tarashansky[1], Stephen R. Quake[1,3,4], Bo Wang[1,5,*]

[1]Department of Bioengineering, Stanford University, Stanford, CA 94305, USA.
[2]Department of Biology, Stanford University, Stanford, CA 94305, USA.
[3]Department of Applied Physics, Stanford University, Stanford, CA 94305, USA.
[4]Chan Zuckerberg Biohub, San Francisco, CA 94158, USA.
[5]Department of Developmental Biology, Stanford University School of Medicine, Stanford, CA 94305, USA.
[#]These authors contributed equally.

Contents

Supplementary Figure 1: Characterization of presumptive progenitor populations.
Supplementary Figure 2: Sequence alignment of *onecut* homologs identified in the *S. mansoni* and *S. mediterranea* genomes.
Supplementary Figure 3: *Sm-oc-1* and *Sm-boule* are coexpressed in *nanos-1*$^+$ cells in testes but only *Sm-boule* is expressed in ovary primordia.
Supplementary Figure 4: RNAi of *irx* and *nr* do not cause any noticeable phenotype.
Supplementary Figure 5: *oc-1* and *boule* RNAi do not change apoptotic activity in testes.
Supplementary Figure 6: *DZIP* RNAi does not cause any noticeable phenotype in either juvenile or adult parasites.
Supplementary Figure 7: RNAi phenotypes of GSC regulators in adult parasites.
Supplementary Figure 8: Single and double RNAi experiments have comparable gene knockdown efficiency.
Supplementary Figure 9: Expression of planarian *onecut* homologs.
Supplementary Figure 10: Targeting non-overlapping regimes in RNAi experiments rules out potential off-target effect.

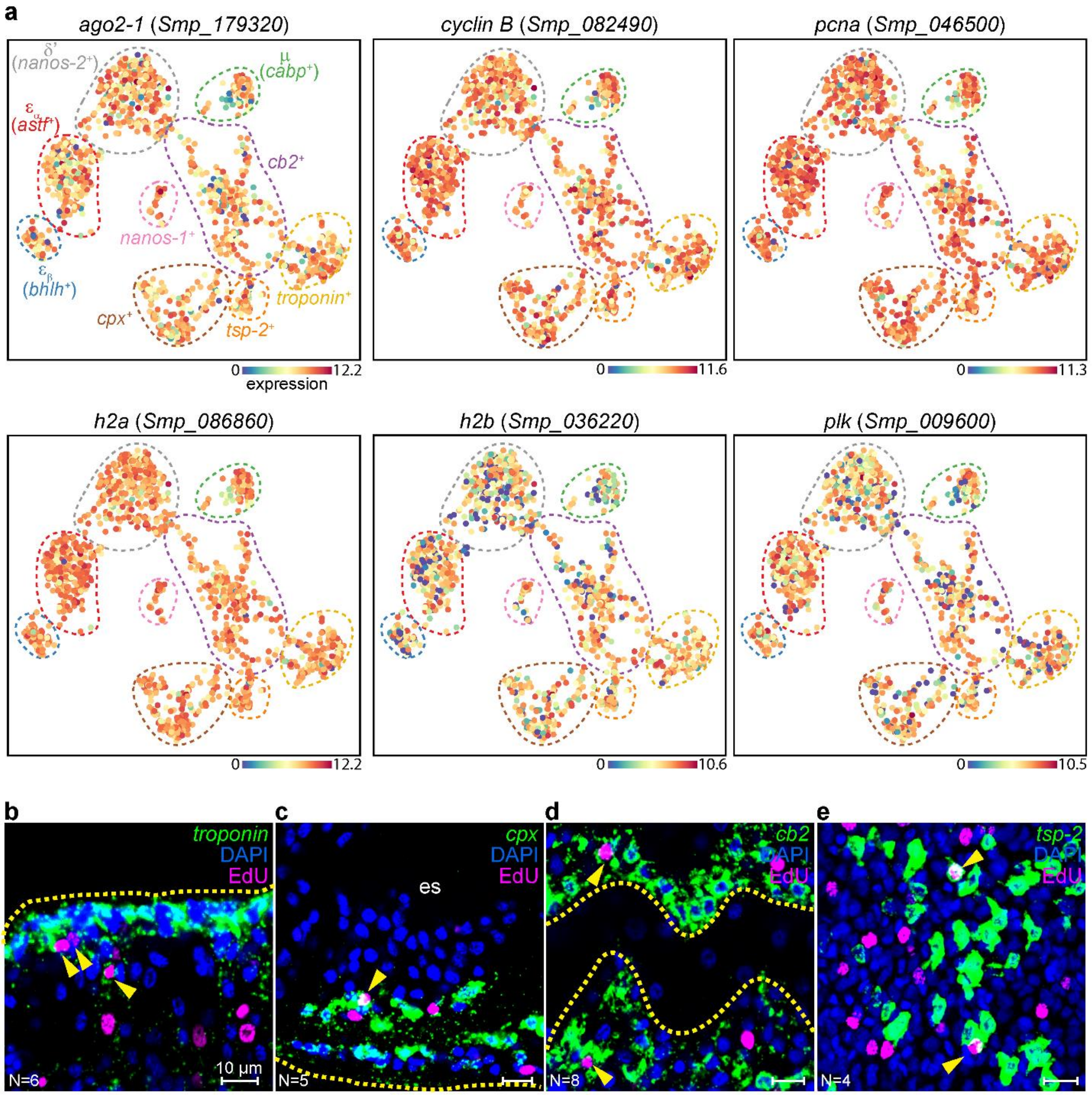

Supplementary Figure 1: Characterization of presumptive progenitor populations.
(a) UMAP projections showing that stem cell markers are ubiquitously expressed at high levels among *ago2-1*$^{+}$ cells, including all the progenitor populations. (b-e) EdU^{+} cells are present within *troponin*$^{+}$ (b), *cpx*$^{+}$ (c), *cb2*$^{+}$ (d), and *tsp-2*$^{+}$ (e) populations, with examples highlighted by arrowheads. Dashed outlines: parasite surface in (b,c) and intestinal branches in (d). “es” denotes esophagus in (c). (e) is imaged at the parasite surface. N: number of samples from two biological replicates showing similar results.

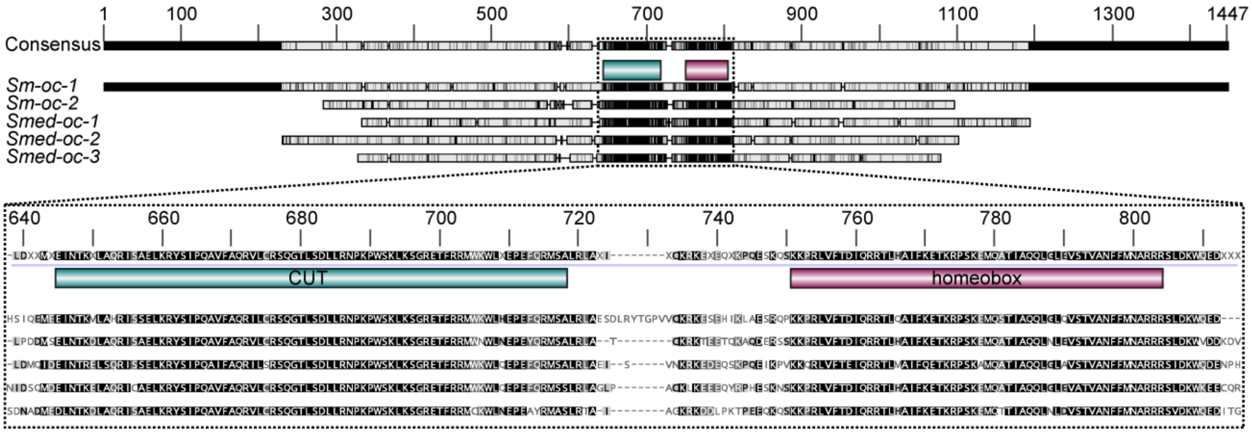

Supplementary Figure 2: Sequence alignment of *onecut* homologs identified in the *S. mansoni* and *S. mediterranea* genomes.

The alignment is performed using the multiple align function in Geneious prime with default settings. *onecut* homologs contain a CUT domain (green) and a homeobox domain (magenta). The domains are annotated by the InterProScan with the application of PfamA on the consensus sequence. Grey scale denotes the similarity of amino acids. Black: 100% similarity; white: less than 60% similarity. *Sm-oc-2* (Smp_013070) is not detectable in juvenile schistosomes by WISH. The gene IDs (PlanMine) of planarian *onecut* homologs are dd_Smes_v1_39638_1_1 (*Smed-oc-1*), dd_Smes_v1_39585_1_2 (*Smed-oc-2*), and dd_Smes_v1_35775_2_1 (*Smed-oc-3*).

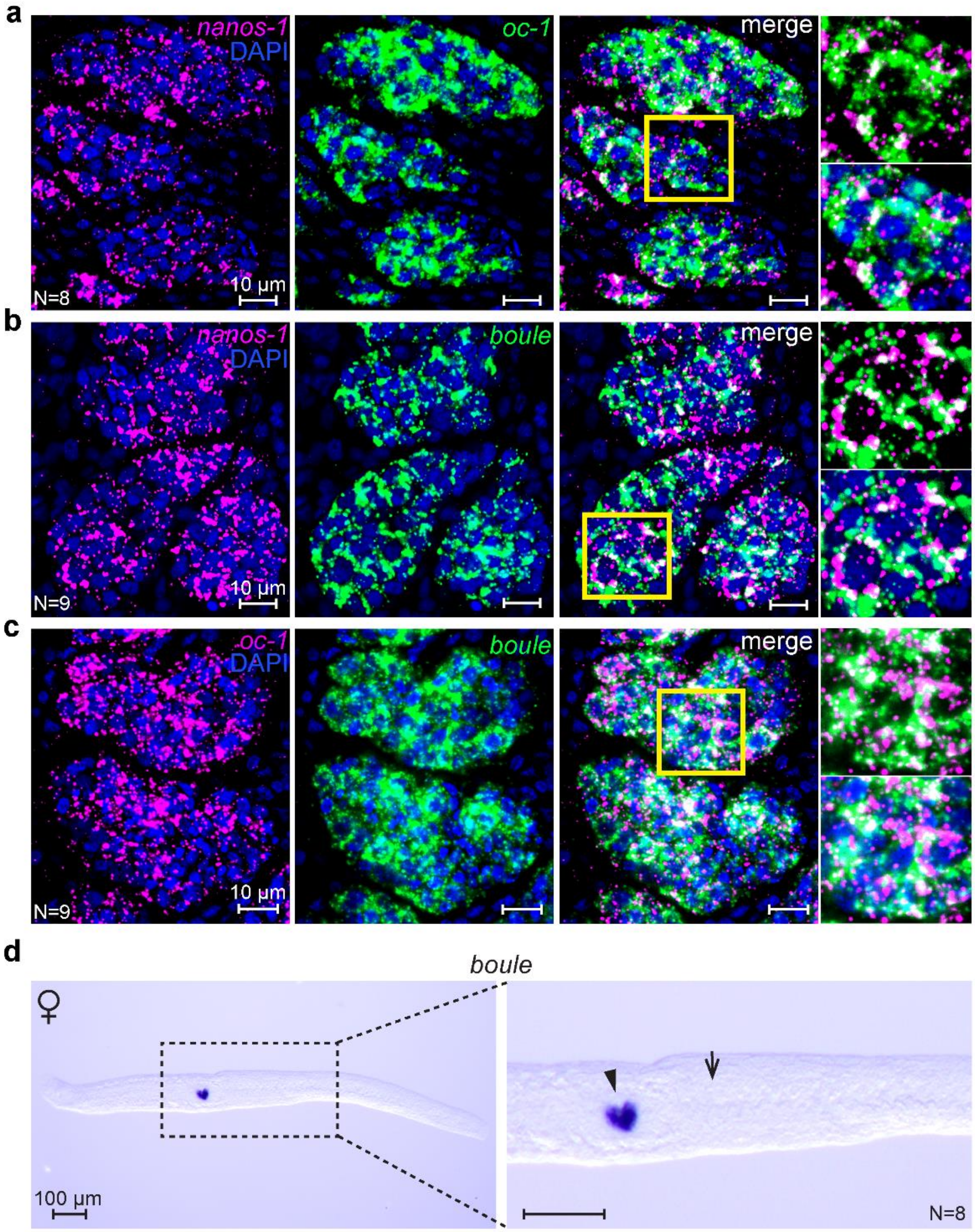

Supplementary Figure 3: *Sm-oc-1* and *Sm-boule* are coexpressed in *nanos-1*$^{+}$ cells in testes but only *Sm-boule* is expressed in ovary primordia.

Double FISH of (a) *nanos-1* and *oc-1*, (b) *nanos-1* and *boule*, (c) *oc-1* and *boule* showing their colocalization in juvenile testis primordia. Magnified views of boxed areas are shown to the right of the merge panel. (d) WISH images of female juveniles showing *boule* expression in ovary primordia (arrowhead), but not in primordial vitellaria (arrow). N: number of samples from two biological replicates showing similar results.

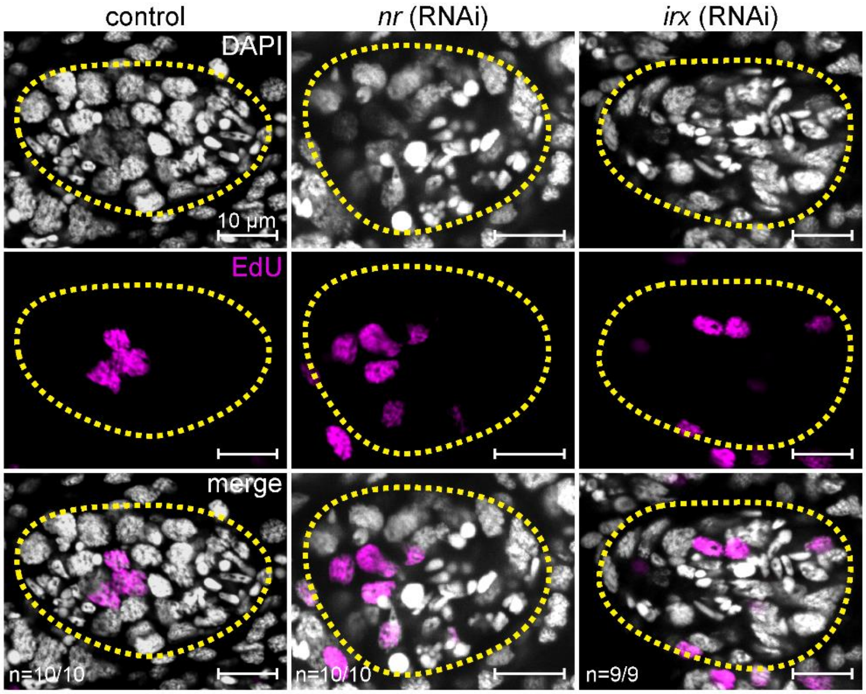

Supplementary Figure 4: RNAi of *irx* and *nr* do not cause any noticeable phenotype. Confocal images of representative individual testis lobules stained with DAPI and EdU after control, *irx*, and *nr* RNAi in juveniles. Dashed circles: testis lobule boundary. n: number of samples exhibiting the reported phenotype out of the total number of samples analyzed. RNAi experiments were repeated on at least two biological replicates.

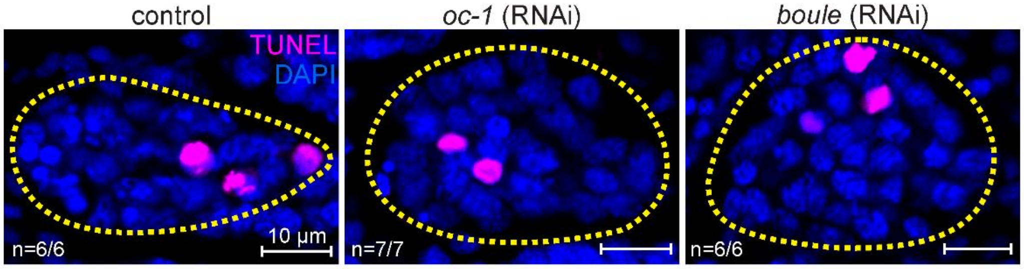

Supplementary Figure 5: *oc-1* and *boule* RNAi do not change apoptotic activity in testes. Confocal images showing individual testis lobules stained by TUNEL to detect apoptotic cells. Note that apoptotic nuclei have the GSC morphology suggesting that GSCs undergo continuous turnover. n: number of samples exhibiting the reported phenotype out of the total number of samples analyzed. RNAi experiments were repeated on at least two biological replicates.

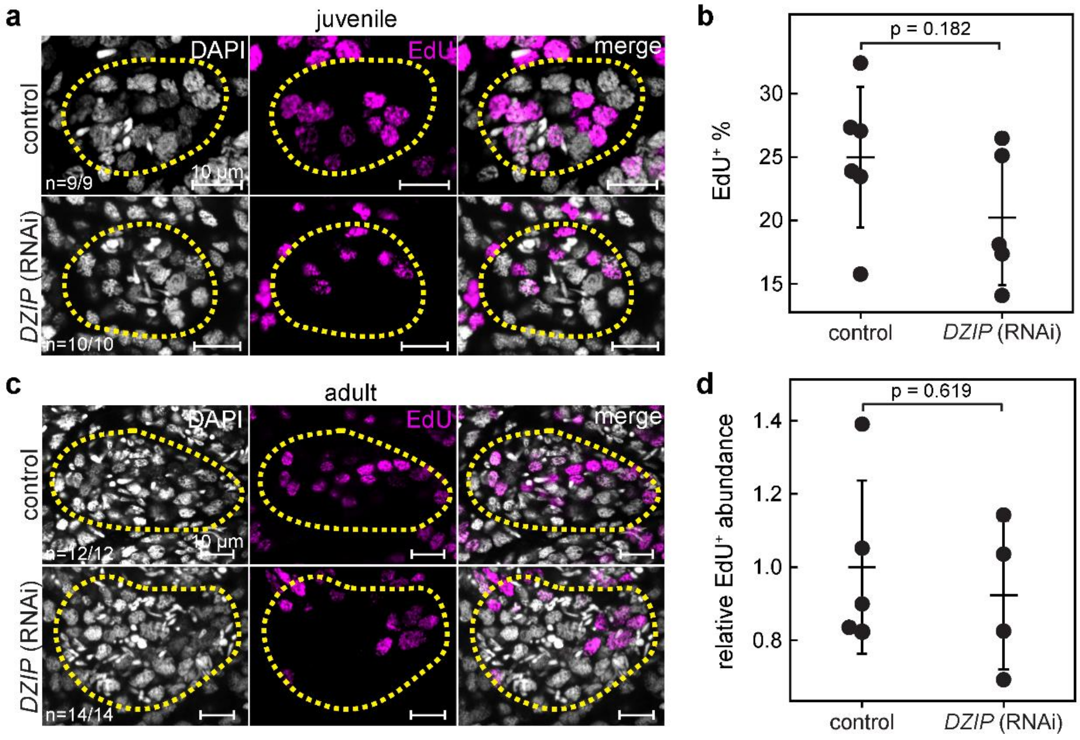

Supplementary Figure 6: *DZIP* RNAi does not cause any noticeable phenotype in either juvenile or adult parasites.

(a) Confocal images showing representative individual testis lobules to compare control and *DZIP* RNAi in juveniles, with the fraction of EdU^+ nuclei quantified in (b). N = 6 (control RNAi); N = 5 (*DZIP* RNAi). Data on adult parasites are reported in (c) with the relative abundance of EdU^+ nuclei per unit image area normalized against the abundance in control parasites shown in (d). N = 5 (control RNAi); N = 4 (*DZIP* RNAi). Dashed circles: testis lobule boundary. In (a,c), n: number of samples exhibiting the reported phenotype out of the total number of samples analyzed. In (b,d), data are presented as average ± standard deviation and p-values are calculated using two-sided Welch's t-test. RNAi experiments were repeated on at least two biological replicates.

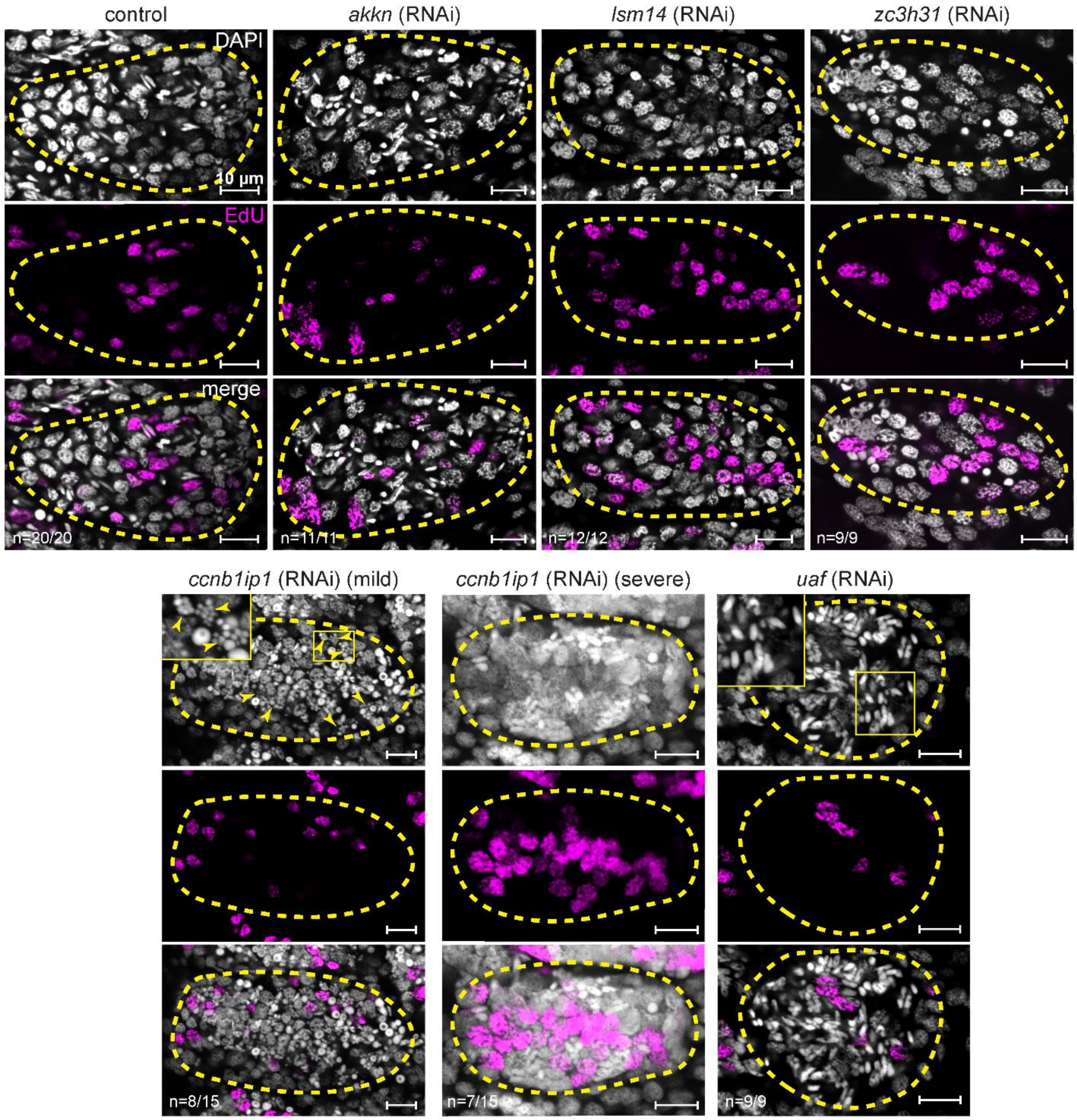

Supplementary Figure 7: RNAi phenotypes of GSC regulators in adult parasites.

Confocal images showing representative individual testis lobules stained by DAPI and EdU in control and *akkn*, *lsm14*, *zc3h31*, *ccnb1ip1*, and *uaf* RNAi. *ccnb1ip1* RNAi can result in both a mild phenotype in which the separation of meiotic nuclei is incomplete (arrowheads), and a severe phenotype, in which nuclei appear to fuse between many cells. Insets: magnified boxed areas. Dashed circles: testis lobule boundary. n: number of samples exhibiting the reported phenotype out of the total number of samples analyzed. RNAi experiments were repeated on at least three biological replicates.

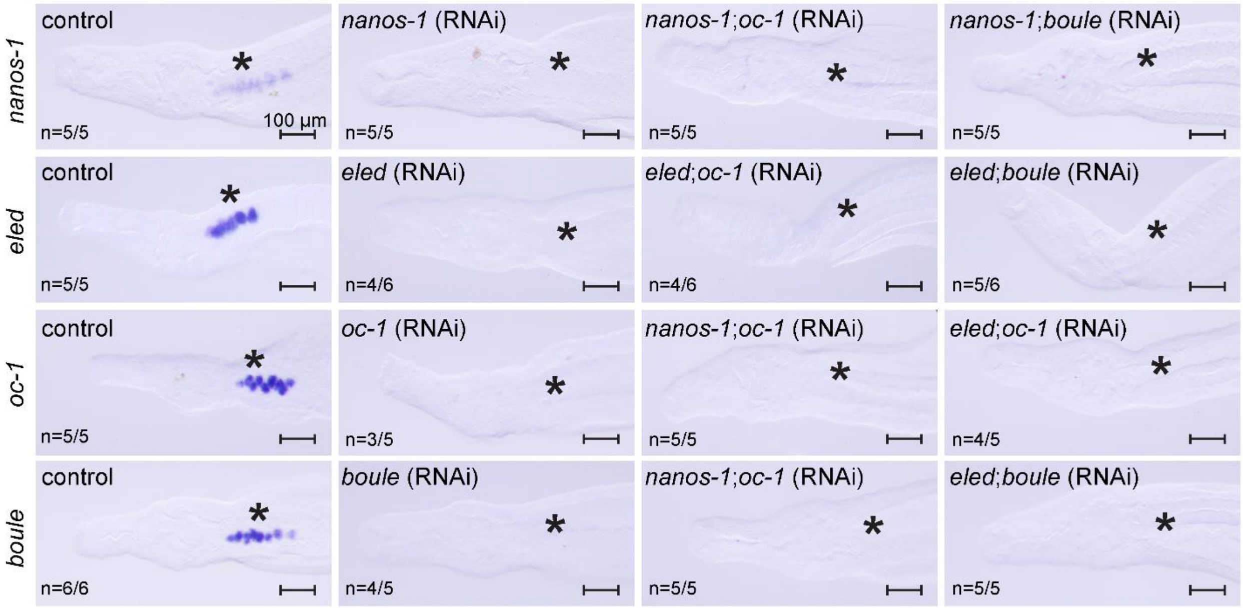

Supplementary Figure 8: Single and double RNAi experiments have comparable gene knockdown efficiency.

WISH experiments are performed in parallel to validate the knockdown of target genes. In all single RNAi and double RNAi experiments, target gene expressions are below the detection limit of the WISH protocol. n: number of samples exhibiting the reported phenotype out of the total number of samples analyzed. Asterisks: testes. RNAi experiments were repeated on at least three biological replicates.

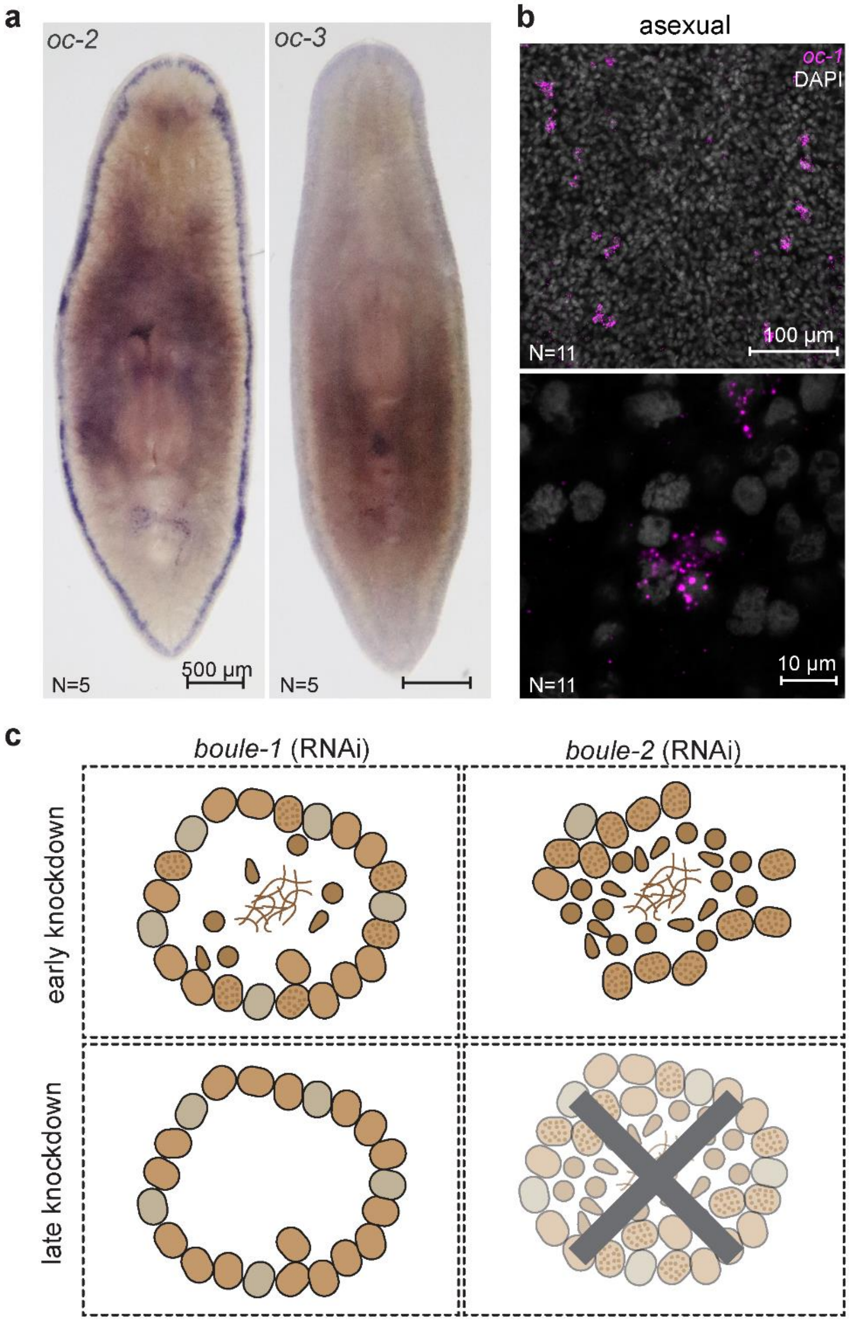

Supplementary Figure 9: Expression of planarian *onecut* homologs.
(a) WISH images showing the expression of *Smed-oc-2* and *Smed-oc-3* in sexually mature planarians.(b) FISH images showing that *Smed-oc-1* is also detected in presumptive GSC clusters in asexual planarians. In (a,b), N: number of samples over two independent experiments showing similar results. (c) Schematics showing the phenotypes observed after *Smed-boule-1* RNAi and *Smed-boule-2* RNAi as reported in ref. 28. After *Smed-boule-1* RNAi, differentiated germ cells are eliminated, whereas *Smed-boule-2* RNAi causes rapid loss of GSCs and spermatogonia and eventually full degeneration of the testes.

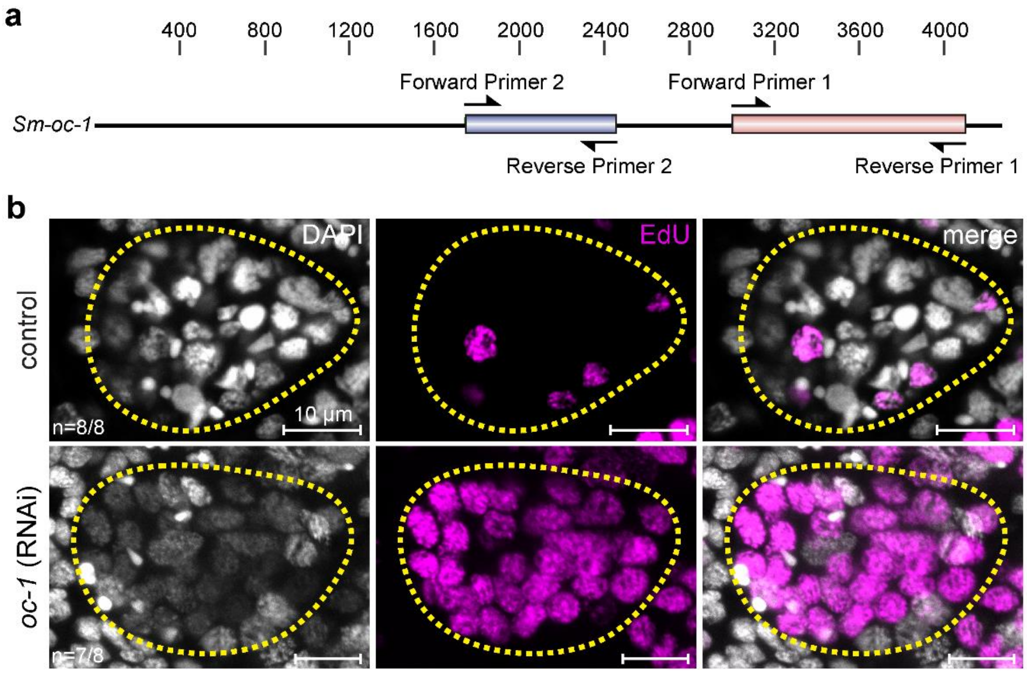

Supplementary Figure 10: Targeting non-overlapping regimes in RNAi experiments rules out potential off-target effect.
(a) As an example, two non-overlapping fragments of *oc-1* are cloned using two different sets of primers and used for dsRNA synthesis. (b) Confocal images of DAPI and EdU stained testes in control and *oc-1* RNAi male juvenile parasites. Dashed circles: testis lobule boundary. In this experiment, the gene fragment was cloned using the primer set 2, whereas for results reported in all other figures the gene fragment was cloned using the primer set 1. The primer sequences are listed in the **Supplementary Data 2**. RNAi experiments using dsRNA synthesized from the two non-overlapping fragments yield identical phenotype. n: number of samples exhibiting the reported phenotype out of the total number of samples analyzed.
