# Supplementary material for: Single-cell analysis of Schistosoma mansoni identifies a conserved genetic program controlling germline stem cell fate
Source: Nat Commun. 2021 Jan 20;12:485. doi: 10.1038/s41467-020-20794-w (PMC7817839; doi:10.1038/s41467-020-20794-w)
Supplement: Supplementary file 3 — Description of Additional Supplementary Files [file 41467_2020_20794_MOESM3_ESM.pdf]

Title: Supplementary Data 1.
Description: List of genes enriched in individual stem cell clusters, along with their mean expression, SAM weight, enriched population, and fold enrichment. Fold enrichment is calculated as the average raw counts within a cluster divided by the average raw counts in all other stem cell clusters. Genes are considered stem cell-specific if there are no differentiated somatic cell types with greater than 1 average log2(CPM).

Title: Supplementary Data 2.
Description: List of genes examined by RNAi. Oligonucleotide sequences used for cloning are also listed.

Title: Supplementary Data 3.
Description: Additional primer sequences for cloning used in this study.
