# Supplementary material for: Single-cell analysis of Schistosoma mansoni identifies a conserved genetic program controlling germline stem cell fate
Source: Nat Commun. 2021 Jan 20;12:485. doi: 10.1038/s41467-020-20794-w (PMC7817839; doi:10.1038/s41467-020-20794-w)
Supplement: Supplementary file 7 — Reporting Summary [file 41467_2020_20794_MOESM7_ESM.pdf]

nature research

Corresponding author(s): Bo Wang

Last updated by author(s): Oct 31, 2020

# Reporting Summary

Nature Research wishes to improve the reproducibility of the work that we publish. This form provides structure for consistency and transparency in reporting. For further information on Nature Research policies, see our Editorial Policies and the Editorial Policy Checklist.

## Statistics

For all statistical analyses, confirm that the following items are present in the figure legend, table legend, main text, or Methods section.

| n/a | Confirmed | |
|---|---|---|
| ☐ | ☒ | The exact sample size ($n$) for each experimental group/condition, given as a discrete number and unit of measurement |
| ☐ | ☒ | A statement on whether measurements were taken from distinct samples or whether the same sample was measured repeatedly |
| ☐ | ☒ | The statistical test(s) used AND whether they are one- or two-sided<br>*Only common tests should be described solely by name; describe more complex techniques in the Methods section.* |
| ☒ | ☐ | A description of all covariates tested |
| ☒ | ☐ | A description of any assumptions or corrections, such as tests of normality and adjustment for multiple comparisons |
| ☐ | ☒ | A full description of the statistical parameters including central tendency (e.g. means) or other basic estimates (e.g. regression coefficient) AND variation (e.g. standard deviation) or associated estimates of uncertainty (e.g. confidence intervals) |
| ☐ | ☒ | For null hypothesis testing, the test statistic (e.g. $F$, $t$, $r$) with confidence intervals, effect sizes, degrees of freedom and $P$ value noted<br>*Give P values as exact values whenever suitable.* |
| ☒ | ☐ | For Bayesian analysis, information on the choice of priors and Markov chain Monte Carlo settings |
| ☒ | ☐ | For hierarchical and complex designs, identification of the appropriate level for tests and full reporting of outcomes |
| ☒ | ☐ | Estimates of effect sizes (e.g. Cohen's $d$, Pearson's $r$), indicating how they were calculated |

*Our web collection on statistics for biologists contains articles on many of the points above.*

## Software and code

Policy information about availability of computer code

| | |
|---|---|
| Data collection | Python 3.6.10, Salmon 0.14.0, scanpy 1.5.1, sam-algorithm 0.7.1, umap-learn 0.3.10, seaborn 0.10.0, scikit-learn 0.22.2, leidenalg 0.7.0 |
| Data analysis | SAM analysis source code can be found at https://github.com/atarashansky/self-assembling-manifold. |

For manuscripts utilizing custom algorithms or software that are central to the research but not yet described in published literature, software must be made available to editors and reviewers. We strongly encourage code deposition in a community repository (e.g. GitHub). See the Nature Research guidelines for submitting code & software for further information.

## Data

Policy information about availability of data

All manuscripts must include a data availability statement. This statement should provide the following information, where applicable:

- Accession codes, unique identifiers, or web links for publicly available datasets
- A list of figures that have associated raw data
- A description of any restrictions on data availability

GEO dataset access number: GSE147355

# Field-specific reporting

# Life sciences study design

All studies must disclose on these points even when the disclosure is negative.

| | |
|---|---|
| Sample size | Our whole body single-cell RNA-seq analysis on schistosome juvenile parasites was the first of its kind; there was no information available to predetermine the sample size . We collected as many cells as practical at the time. For the RNAi experiments, the sample size was determined based on our previous studies (e.g., Wang et al. eLife, 2018, 7:e35449) in order to obtain sufficient statistics. All RNAi experiments on schistosomes were done on at least two biological replicates, with each containing parasites collected from a separate batch of infected mice, and each biological replicate included at least two technical replicates, each processed separately but in parallel. All RNAi experiments on planarians were done on at least two biological replicates, each using a separate batch of worms and independently synthesized dsRNA. |
| Data exclusions | Quality filtering of single-cell transcriptional profiles is explicitly described in the methods. Due to limitations in long-term in vitro culture of the parasites, filtering of animals with deteriorated physiology and retarded development in RNAi experiments is explicitly described in the text and methods. |
| Replication | Experimental findings were reliably reproduced by technical and biological replicates. This information is extensively described in the text, the methods, and the figure captions. |
| Randomization | Each batch of parasites were collected from 5-20 mice, and parasites collected from different mice were mixed and used randomly in various experiments. |
| Blinding | When evaluating phenotypic changes after RNAi, researchers were blind to genes that had been knocked down. |

# Reporting for specific materials, systems and methods

We require information from authors about some types of materials, experimental systems and methods used in many studies. Here, indicate whether each material, system or method listed is relevant to your study. If you are not sure if a list item applies to your research, read the appropriate section before selecting a response.

## Materials & experimental systems

| n/a | Involved in the study | |
|---|---|---|
| ☐ | ☒ | Antibodies |
| ☒ | ☐ | Eukaryotic cell lines |
| ☒ | ☐ | Palaeontology and archaeology |
| ☐ | ☒ | Animals and other organisms |
| ☒ | ☐ | Human research participants |
| ☒ | ☐ | Clinical data |
| ☒ | ☐ | Dual use research of concern |

## Methods

| n/a | Involved in the study | |
|---|---|---|
| ☒ | ☐ | ChIP-seq |
| ☒ | ☐ | Flow cytometry |
| ☒ | ☐ | MRI-based neuroimaging |

## Antibodies

| | |
|---|---|
| Antibodies used | Anti-Digoxigenin-POD, Fab fragments: Roche, 11207733910<br>Anti-Fluorescein-POD, Fab fragments: Roche, 11426346910<br>Anti-Digoxigenin-AP, Fab fragments: Roche, 11093274910<br>Peroxidase AffiniPure Goat Anti-Rabbit IgG (H+L): Jackson ImmunoResearch, 111-035-003 |
| Validation | Multiple lots were used, and results were consistent across lots. These antibodies are subjected rigorous quality control using well-characterized mRNAs as target. |

## Animals and other organisms

Policy information about studies involving animals; ARRIVE guidelines recommended for reporting animal research

| | |
|---|---|
| Laboratory animals | Mouse host: Swiss webster<br>Schistosoma mansoni: Strain NMRI<br>Planarian: asexual Schmidtea mediterranea (CIW4 strain) and sexual strain Schmidtea mediterranea |
| Wild animals | This study did not involve wild animals. |
| Field-collected samples | This study did not involve samples collected from the field. |
| Ethics oversight | All mice were treated according to Animal Care and Use Committee (IACUC) protocols. Procedures of handling animals are described explicitly in the text and methods. |

Note that full information on the approval of the study protocol must also be provided in the manuscript.
